# Microdecompression versus Open Laminectomy and Posterior Stabilization for Multilevel Lumbar Spine Stenosis: A Randomized Controlled Trial

**DOI:** 10.1155/2019/7214129

**Published:** 2019-11-07

**Authors:** Sherwan A. Hamawandi, Injam Ibrahim Sulaiman, Ameer Kadhim Al-Humairi

**Affiliations:** ^1^Department of Orthopaedics, College of Medicine, Hawler Medical University, Erbil, Iraq; ^2^Department of Neurosurgery, College of Medicine, Hawler Medical University, Erbil, Iraq; ^3^Dept. of Community Medicine, College of Medicine, University of Babylon, Hilla, Iraq

## Abstract

**Background:**

Lumbar spinal stenosis most often results from a gradual, degenerative ageing process. Open or wide decompressive laminectomy was formerly the standard treatment. However, in recent years, a growing tendency towards less invasive decompressive procedures has emerged. The purpose of this study was to compare the results of microdecompression with those of open wide laminectomy and posterior stabilization for patients with symptomatic multilevel lumbar spinal stenosis who failed to respond to conservative treatment.

**Methods:**

This randomized controlled study was conducted between January 2016 and October 2018. One hundred patients were involved in this study. All these patients suffered from radicular leg pain with MRI features of multilevel lumbar spinal stenosis and were treated by conservative treatment of medical treatment and physiotherapy without benefit for 6 months. Those patients were divided into two groups: Group A, 50 microdecompression, and Group B, 50 patients who were treated by open wide laminectomy and posterior stabilization. Both groups of patients were followed up with ODI (Oswestry disability index) and VAS (visual analogue score) for the back and leg pain for one year.

**Results:**

The results showed that both groups got significant improvement regarding the Oswestry disability index. Regarding back pain, there was a significant improvement in both groups with better results in group A due to minimal tissue injury as the advantage of the minimal invasive technique. In both groups, there was marked improvement of radicular leg pain postoperatively.

**Conclusions:**

Both microdecompression and wide open laminectomy with posterior stabilization were effective in treatment of multilevel lumbar spinal stenosis with superior results of microdecompression regarding less back pain postoperatively with less blood loss and soft tissue dissection. Clinical trial number: NCT04087694.

## 1. Introduction

Stenosis of the lumbar spine is an extremely widespread disorder that frequently arises from a gradual degenerative ageing progression [1]. The clinical condition of the stenosis is characterized by low back pain and pain and numbness in the legs, and it is a common cause of weakened walking and inability in elderly people (≥60 years). It is the most frequent indication for spinal surgery in the elderly [2]. Management of spinal stenosis can be challenging and needs the incorporation of patients' symptoms, clinical results, and diagnostic imaging. There is rising evidence that decompressive surgery offers a priority over nonsurgical management for particular patients with continual severe signs [3]. Presently, it is normally accepted that surgery is designated if conservative or nonsurgical management fails. Development in radiating pain, neurogenic claudication, functional position, and quality of life are the major treatment aims. Open laminectomy, often combined with medial facetectomy and foraminotomy, has conventionally been the typical therapy in patients without instability [4, 5]. In recent years, less invasive measures have developed [6, 7] and microdecompression through smaller incisions is often achieved. Decompressive laminectomy is widely used to treat LSS. Although satisfactory surgical outcomes have been reported using this technique, instability following the procedure is one of the greatest concerns amongst surgeons as it may cause deterioration of symptoms [4] In a research conducted in 2005, unilateral microdecompression for bilateral decompression and bilateral microdecompression were found to be hopeful therapy alternatives when compared with open decompressive laminectomy [4]. Subsequently, unilateral and bilateral microdecompression have been adopted by several spine surgeons, and as is the case in Norway, often among neurosurgeons than orthopaedic surgeons. However, there is still a necessity to assess the benefits and risks of different decompressive surgical measures for lumbar spinal stenosis [8, 9].

The present study aimed to compare the results of microdecompression for multilevel lumbar spine stenosis with those of open laminectomy and posterior stabilization regarding the Oswestry disability index and visual analogue score for back pain and leg pain.

## 2. Methods

The protocol of this study was reviewed and approved by the research ethics committee in our university. Written informed consents were obtained from all patients. This study is a randomized controlled trial. One hundred patients were involved in this study from January 2016 to October 2018. All these patients suffered from back pain of different degrees with spinal claudication and were treated by conservative treatment of medical treatment and physiotherapy without benefit for 6 months at least. MRI of the lumbosacral spine showed multilevel spinal stenosis L3-S1, and all patients were assessed clinically and radiologically. All patients underwent dynamic flexion and extension lumbosacral plain x-ray to exclude any instability.

The patients were divided into two groups according to the ODD and EVEN number on receiving of the patients: Group A, 50 patients who were treated by microdecompression, and Group B, 50 patients who were treated by open laminectomy and posterior stabilization with pedicle screws from L3 to S1 levels.

The two groups of patients were operated by one team which consisted of one orthopaedic surgeon and one neurosurgeon. The instability was assessed by dynamic X-ray, and those cases with instability were excluded. Cases with decreased disc height and disc degeneration of significant degrees were not involved in this study. All patients in this study suffered from radiculopathy as the primary complain, and those cases with only back pain (discogenic pain) are not involved in this study.

All these patients were assessed and followed up by ODI preoperatively and 1 month postoperatively and VAS for back pain and leg pain preoperatively, in addition to 1, 6, and 12 months postoperatively.

### 2.1. Exclusion Criteria

Exclusion criteria of the present study include smoking, diabetic patients, previous spinal surgery, any neuromuscular disorder like poliomyelitis, vertebral instability proved by dynamic plain radiographs, and patients with significant loss of disc height and degeneration.

### 2.2. Data Analysis

Statistical analysis was carried out using SPSS version 21 for Windows (SPSS Inc., Chicago, IL, USA). Categorical variables were presented as frequencies and percentages. Continuous variables were presented as Mean ± SD. The student's *t*-test was used to compare means between two groups. The Mann–Whitney *U* test was used to compare two groups when the variable was not normally distributed. The paired *t*-test was used to compare means for paired reading. The Pearson's chi-square test (χ^2^) was used to find the association between categorical variables. A *P* value of ≤0.05 was considered as significant.

### 2.3. Surgical Procedures

#### 2.3.1. Group A

Under general anesthesia, with supine position and flexion of both hips and knees by pillows, and with the aid of a microscope, midline incision was done after determination of the spinal levels by fluoroscopy with cautery. The deep fascia was opened, and paravertebral muscles were retracted laterally to expose the lamina of L5 on the symptomatic side. Then, by high speed drill the lamina was thinned by passing a hook under the lamina and retracting the ligamentum flavum; then, by using tenotome the ligamentum falvum was incised over the hook; then, by karyson the ligamentum flavum was removed to expose the dura and the nerve root on that side; foraminotomy was performed; and then, the microscope was tilted 15 degrees, and the bed of the patient was tilted 15 degrees. Therefore, we directed the microscope on the contralateral side to remove a part of the lamina and the ligementum flavum to decompress the contralateral nerve root; then, hemostasis was performed starting with proximal level (L4) and then (L3) with the same technique but on the alternating way. After securing hemostasis, the surgical wound was closed in layers with no drain. The patients are mobilized after 6–8 hours after operation.

#### 2.3.2. Group B

Under general anesthesia, with supine position and flexion of both hips and knees by pillows, midline incision was performed after determination of the target levels from L3 to S1. By cautery, the deep fascia was opened, and the paravertebral muscles were retracted to expose the laminae from L3 to L5. Insertion of pedicle screws from L3 to S1 (the stenosed levels) and wide laminectomy were performed to the stenosed levels with decompression of the nerve roots and then hemostasis secured. The rodes were inserted with consideration of lumbosacral lordosis; decortication was performed, and the bone grafts were put posterolaterally from the removed spinous processes and laminae. The surgical wound was closed in layers with drain which was removed the next day, and the patient started mobilization the next day postoperatively.

## 3. Results

Table 1 shows the distribution of patients according to sociodemographic characteristics (including age and gender).

Table 2 shows the mean differences of age between study groups including Group A patients who underwent microdecompression surgery and Group B patients who underwent open decompression and spine fixation. There were no significant differences between means of age between these two groups.

Table 3 shows the association between gender and study group including Group A patients who underwent microdecompression surgery and Group B patients who underwent open decompression and spine fixation. There was no significant association between gender and study group.

**Figure 1:**
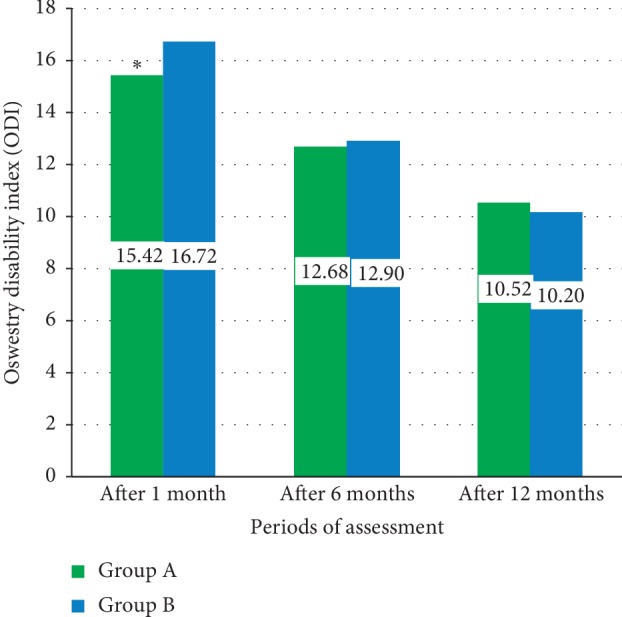
The mean differences of the postoperative Oswestry disability index (ODI) between study groups.

Figure 1 shows the mean differences of the postoperative Oswestry disability index (ODI) between study groups including Group A patients who underwent microdecompression surgery and Group B patients who underwent open decompression and spine fixation. There were significant differences between means of the ODI between these two groups after one month (*P*=0.001^*∗*^), while there were nonsignificant differences between two groups after six and twelve months of operation (*P*=0.421 and *P*=0.57).

**Figure 2:**
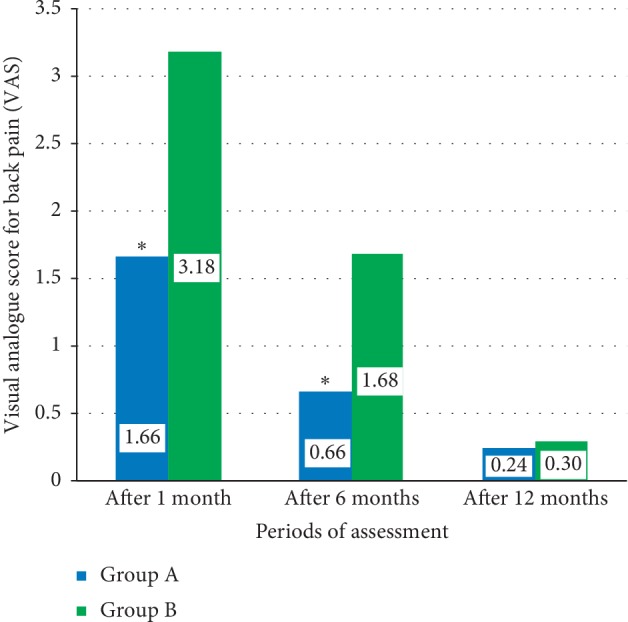
The mean differences of the postoperative visual analogue index (VAS) for back pain between study groups.

Figure 2 shows the mean differences of the postoperative visual analogue score (VAS) for back pain between study groups including Group A patients who underwent microdecompression surgery and Group B patients who underwent open decompression and spine fixation. There were significant differences between means of VAS for back pain between these two groups after one month and six months (*P* < 0.001^*∗*^, *P* < 0.001^*∗*^), while there were no significant differences between means of VAS for back pain between these two groups after 12 months (*P*=0.524).

**Figure 3:**
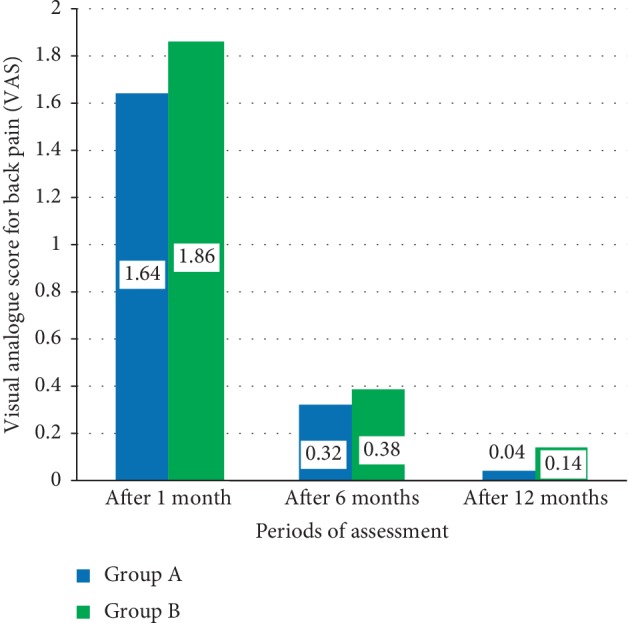
The mean differences of the postoperative visual analogue index (VAS) for leg pain between study groups.

Figure 3 shows the mean differences of the postoperative visual analogue score (VAS) for leg pain between study groups including Group A patients who underwent microdecompression surgery and Group B patients who underwent open decompression and spine fixation. There were no significant differences between means of VAS for leg pain between these two groups after 1, 6, and 12 months (*P*=0.618, *P*=0.604, and *P*=0.23, respectively).

Table 4 shows the mean differences of ODI, VAS for back pain, and VAS for leg pain between preoperative and postoperative assessments three times after 1, 6, and 12 months for group A patients who underwent microdecompression surgery.

Table 5 shows the mean differences of ODI, VAS for back pain, and VAS for leg pain between preoperative and postoperative assessments three times after 1, 6, and 12 months for group B patients who underwent open decompression and spine fixation.

Table 6 shows the mean differences of operation time (in minutes) and amount of blood lost (in ml) between study groups including Group A patients who underwent microdecompression surgery and Group B patients who underwent open decompression and spine fixation. There were significant differences between means of operation time and amount of blood lost between these two groups.

Table 7 shows the association between the cost of operation and study group including Group A patients who underwent microdecompression surgery and Group B patients who underwent open decompression and spine fixation. There was significant association between the cost of operation and study groups.

## 4. Discussion

In our study, we found that both groups got significant improvement regarding the Oswestry disability index (Table 1). Regarding back pain, there was a significant improvement in both groups with better results in group A due to minimal tissue injury as the advantage of minimal invasive technique (Table 2). In both groups, there was a marked improvement of radicular leg pain postoperatively (Table 3). In comparison with previous observational studies [4, 6, 7], secondary outcome analyses showed a major improvement in health-related quality of life in both treatment groups. Although results at one year were extremely similar, patients in the microdecompression group had shorter hospital stays than patients who went through laminectomy.

This result was reliable using various policies for analyzing data. A possible clarification is that microdecompression decreases surgical trauma, permitting early mobiliation after surgery. Nevertheless, it is also probable that surgical units adapting to least invasive procedures will be prone towards shorter hospital stays, taking different practices for postoperative mobilization, pain management, and hospital discharge. Conventional laminectomy with removal of posterior bony and ligamentous structures has been the gold standard of surgical treatment for decades. Although postoperative development of segmental instability is a multifactorial problem, unnecessary damage to anatomic structures, which stabilize the functional spinal unit, has always been a problem with this technique [8–11]. Moreover, the fact that the spinal canal is exposed more than what would be necessary just for a decompression increases the contact surface between paravertebral muscles and the dura is one of the reasons for extensive scar tissue formation and epidural fibrosis following conventional laminectomy, which may lead to tethering of the cauda equina and radicular symptoms [9, 12, 13]. Microsurgical crossover decompression through a unilateral approach significantly minimizes these problems [14–18]. The muscles are retracted only on one side, and the area of the spinal canal, which is exposed to the surrounding tissue, remains small. This reduces the area of potential scar formation. Moreover, the integrity of the contralateral facet joint remains nearly completely intact.

All cases in group A (who underwent microdecompression) had no instability as instability was considered as an exclusion criterion (the patients with vertebral instability are not included in our study, whether group A or group B).

## 5. Conclusion

Both microdecompression and open laminectomy with posterior stabilization were effective treatment methods for lumbar spinal stenosis regarding leg pain with less postoperative back pain in the group of microdecompression with less operative time, less blood loss, and less cost.

## Figures and Tables

**Table 1:** The distribution of patients according to sociodemographic characteristics.

Sociodemographic variables		
Age (years)	(55.9 ± 8.03)	(37.0 – 74.0)

Gender		
Male	35	35%
Female	65	65%
Total	100	100.0%

**Table 2:** The mean differences of age between study groups.

Study variables	Study groups	*N*	Mean	SD	*t*-test	*P* value
Age (years)	Group A	50	56.60	7.79	0.87	0.386
	Group B	50	55.20	8.28		

**Table 3:** The association between gender and study group.

Study variables	Study group		*χ*2	*P* value
	Group A	Group B		
Gender				
Male	16 (32.0)	19 (38.0)	**0.396**	**0.529**
Female	34 (68.0)	31 (62.0)		
Total	50 (100.0)	50 (100.0)		

^*∗*^ *P* value ≤0.05 was significant.

**Table 4:** The mean differences of ODI, VAS for back pain, and VAS for leg pain between preoperative and postoperative assessments three times.

Study variables	Periods of assessment	*N*	Mean	SD	Paired *t*-test	*P* value
ODI	Preoperative ODI	50	71.30	1.69	**94.57**	**<0.001** ^*∗*^
	1 month postoperative ODI	50	15.42	3.59		
	Preoperative ODI	50	71.30	1.69	**177.31**	**<0.001** ^*∗*^
	6 months postoperative ODI	50	12.68	1.44		
	Preoperative ODI	50	71.30	1.69	**237.4**	**<0.001** ^*∗*^
	12 months postoperative ODI	50	10.52	0.88		

VAS for back pain	Preoperative VAS for back pain	50	5.22	0.70	**19.66**	**<0.001** ^*∗*^
	1 month postoperative VAS for back pain	50	1.66	1.08		
	Preoperative VAS for back pain	50	5.22	0.70	**42.41**	**<0.001** ^*∗*^
	6 month postoperative VAS for back pain	50	0.66	0.55		
	Preoperative VAS for back pain	50	5.22	0.70	**56.58**	**<0.001** ^*∗*^
	12 month postoperative VAS for back pain	50	0.24	0.43		

VAS for leg pain	Preoperative VAS for leg pain	50	9.86	0.35	**71.28**	**<0.001** ^*∗*^
	1 month postoperative VAS for leg pain	50	1.64	0.80		
	Preoperative VAS for leg pain	50	9.86	0.35	**99.72**	**<0.001** ^*∗*^
	6 month postoperative VAS for leg pain	50	0.32	0.55		
	Preoperative VAS for leg pain	50	9.86	0.35	**178.92**	**<0.001** ^*∗*^
	12 month postoperative VAS for leg pain	50	0.04	0.19		

**Table 5:** The mean differences of ODI, VAS for back pain, and VAS for leg pain between preoperative and postoperative assessments three times.

Study variables	Periods of assessment	*N*	Mean	SD	Paired *t*-test	*P* value
ODI	Preoperative ODI	50	72.24	2.38	**84.07**	**<0.001** ^*∗*^
	1 month postoperative ODI	50	16.72	4.07		
	Preoperative ODI	50	72.24	2.38	**155.4**	**<0.001** ^*∗*^
	6 months postoperative ODI	50	12.90	1.26		
	Preoperative ODI	50	72.24	2.38	**179.73**	**<0.001** ^*∗*^
	12 months postoperative ODI	50	10.20	1.62		

VAS for back pain	Preoperative VAS for back pain	50	5.14	0.90	**11.28**	**<0.001** ^*∗*^
	1 month postoperative VAS for back pain	50	3.18	1.15		
	Preoperative VAS for back pain	50	5.14	0.90	**23.21**	**<0.001** ^*∗*^
	6 month postoperative VAS for back pain	50	1.68	0.84		
	Preoperative VAS for back pain	50	5.14	0.90	**32.99**	**<0.001** ^*∗*^
	12 month postoperative VAS for back pain	50	0.30	0.50		

VAS for leg pain	Preoperative VAS for leg pain	50	9.86	0.35	**77.65**	**<0.001** ^*∗*^
	1 month postoperative VAS for leg pain	50	1.86	0.78		
	Preoperative VAS for leg pain	50	9.86	0.35	**103.68**	**<0.001** ^*∗*^
	6 month postoperative VAS for leg pain	50	0.38	0.60		
	Preoperative VAS for leg pain	50	9.86	0.35	**128.21**	**<0.001** ^*∗*^
	12 month postoperative VAS for leg pain	50	0.14	0.45		

**Table 6:** The mean differences of operation time and amount of blood lost between study groups.

Study variables	Study groups	*N*	Mean	SD	*t*-test	*P* value
Operation time (minutes)	Group A	50	118.10	9.30	−38.78	<0.001^*∗*^
	Group B	50	178.00	5.71		

Blood lost (ml)	Group A	50	77.50	9.54	−64.03	<0.001^*∗*^
	Group B	50	308.20	23.62		

**Table 7:** The association between the cost of operation and study groups.

Study variables	Study group		Total	*χ* ^2^	*P* value
	Group A	Group B			
Cost of operation					
4500$	33 (100.0)	0 (0.0)	33 (100.0)	**65.02**	**<0.001** ^*∗*^
6000$	0 (0.0)	32 (100.0)	32 (100.0)		
Performed in a governmental hospital (free)	17 (48.6)	18 (51.4)	35 (100.0)		
Total	50 (100.0)	50 (100.0)	100 (100.0)		

^*∗*^ *P* value ≤0.05 was significant.

## Data Availability

The datasets supporting the conclusions of this article are included within the article.
